# Epilithic Algae from Seven Megaliths in the Vicinity of Topolovgrad (Haskovo District, Southeast Bulgaria)

**DOI:** 10.3390/life15091451

**Published:** 2025-09-16

**Authors:** Maya Petrova Stoyneva-Gärtner, Miroslav Ivov Androv, Blagoy Angelov Uzunov, Kristian Rosenov Ivanov, Georg Gärtner

**Affiliations:** 1 Department of Botany, Faculty of Biology, Sofia University “St. Kliment Ohridski”, 8 Blvd. Dragan Tsankov, 1164 Sofia, Bulgaria; mstoyneva@uni-sofia.bg (M.P.S.-G.); androv@uni-sofia.bg (M.I.A.); kristianri@uni-sofia.bg (K.R.I.); 2 Institute of Botany, Innsbruck University, Sternwartestrasse 15, 6020 Innsbruck, Austria; georg.gaertner@uibk.ac.at

**Keywords:** algal diversity, Cyanoprokaryota, Chlorophyta, global warming, historical monuments, Ochrophyta, Streptophyta, threatened species, toxic algae

## Abstract

The present work focuses on seven megaliths sampled for the first time as a continuation of our studies on the biodiversity of algae on megaliths in Bulgaria. A total of 55 species from four divisions were identified (Chlorophyta was the richest with 31 species), of which 14 species are new for Bulgaria. Two species were of conservation concern, but six were potential toxin producers that could affect the health of visitors to the megaliths. Despite the general granitic character and relatively close location of the studied sites, their floristic similarity was low, with most algae (51 species) being rarely distributed (except *Stichococcus bacillaris* and *Mychonastes homosphaera*). The largest cult complex Paleokastro, furthest from populated areas, had the highest species diversity (22 species), while the lowest (8 species) was found in the highly exposed Kalinkin Kamuk, located in a village. The NMDS analysis tool showed the existence of four distinct ecological groups and that temperature and light are the most important drivers for the distribution of the epilithic algae on the investigated megaliths. Correlations with temperatures estimated to identify potential indicators or future survivors under global warming conditions were low, except for the Streptophyta. It was represented by four species of the genus *Klebsormidium*, which is known for its high ecological tolerance and drought resistance. Extending the studies on lithophytic algae to megaliths is important for a better knowledge of their biodiversity and ecology, but also for the protection of megaliths and for understanding the impact of climate change on these fragile monuments.

## 1. Introduction

Organisms on Earth, and algae in particular, inhabit two main environments—aquatic and terrestrial. The latter include a wide variety of solid substrates: soils of different composition and structure, the surfaces of rocks and stones, tree bark or man-made structures such as roofs, fences, building facades, cultural monuments and various other substrates, even glass [1,2]. Terrestrial algae coined with the terms aeroterrestrial or subaerial [1,2,3,4,5,6,7,8], thrive under much more uncertain and variable conditions than in the aquatic environment. As they are completely dependent on local climatic conditions, they all suffer from the same daily and seasonal hardships, such as: (a) general shortage of water, accompanied by periodic wetting and gradual drying; (b) sharp fluctuations in temperature amplitudes; (c) strong and prolonged solar radiation, often accompanied by direct exposure to ionising ultraviolet (UV) radiation; (d) possible grazing by other organisms; and (e) deficiency or almost total absence of the main biogenic elements, such as nitrogen and phosphorus [9,10,11,12]. As a result of these volatile and highly variable external factors, aeroterrestrial algae have developed a variety of morphological and biochemical adaptations to cope with natural challenges. Their plasticity allows them to colonise some of the most challenging and harsh habitats on Earth—from humid tropical forests to completely arid deserts, snowy alpine regions and the permafrost of the poles (e.g., [13,14]). Subaerial algae are increasingly perceived as extremophilic organisms that can be resistant to other conditions on the planet [15,16]. In addition, their high ecological tolerance makes them sought-after symbionts for organisms from almost all taxonomic groups from the origin of life on Earth to the present day [17]. All these properties place them at the centre of modern biotechnological research and innovative technologies affecting almost all areas of human life [18,19,20,21,22,23] and suggest their increasing role in space exploration [15,16].

While the negative role of subaerial algae as biological corrosion and weathering agents is widely recognized (e.g., [24,25,26,27,28,29,30]), there are conflicting data on their positive protective effect on building materials (for details see [26]). Given this importance for surface structures and their role as pioneer colonisers that enhance further biological development and increase biodiversity [31,32], the growing interest in these particular algae is understandable. Although studies on different substrates and regions are increasing rapidly, we are still far from complete knowledge of their biodiversity. This also concerns the rock inhabitants, lithophytes [5,6,7] and especially those developing on cultural and historical monuments [33,34,35,36,37,38,39,40,41,42,43,44] and is particularly true for the algae growing on ancient megaliths [27,45]. Although the role of these unique historical sites as scientific challenges and tourist magnets in cultural landscapes has been recognized [46,47], the life on their surfaces is still poorly explored [45]. Also, very little is known about the effects of global warming on historical monuments and their algal growth [26,48].

The first study of selected megaliths in Bulgaria revealed not only their rich algal diversity (91 taxa), which includes some globally rare and endangered species, but also the potential risk of their toxic algal inhabitants to the health of megalith visitors [45]. Furthermore, the estimated correlations between total biodiversity and algae from different taxonomic groups with temperature and its extremes in the same publication demonstrated the untapped potential of megalithic algae as indicators of climate-induced change.

The present work is a continuation of this first study and also looks for potential survivors of global warming. Considering the differences in the algal composition of the megaliths from different parts of the large district of Haskovo (5533.292 km^2^ or about 5% of the Bulgarian territory) and their low floristic similarity [45], the present study focuses on a much smaller area (limited to the surroundings of the town of Topolovgrad in the same district) with the idea of comparing the diversity of closer megaliths. It provides data on the biodiversity of algae from seven megaliths in Bulgaria, sampled for the first time, and shows the potential of lithophytes from these ancient structures in the search for indicators and sustainable solutions to climate-related problems we face as a society. The latter effect is enhanced by the known ability of algae to sequester carbon (they are up to 50 times more efficient than higher plants) and thus mitigate greenhouse gas emissions, contributing to the fight against climate change [49,50,51,52].

## 2. Materials and Methods

**Figure 1:**
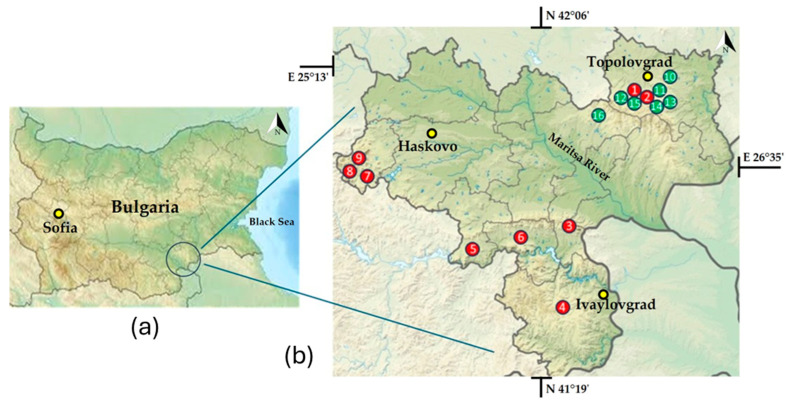
Physical–geographical map of Bulgaria (**a**) with enlarged map of the Haskovo district and indication of the locations of the megaliths (**b**). The green dots represent the seven megaliths discussed in this article, sampled between 28 and 30 June 2023 and located in the vicinity of the town of Topolovgrad. The red dots represent the more widely distributed megaliths throughout the Haskovo district that we sampled between 14 and 18 July 2022 [45]: 1—Tsarski Dolmen, 2—Evdzhika, 3—Gluhite Kamuni, 4—Plevun, 5—Kovan Kaya, 6—Cromleh, 7—Angel Voyvoda, 8—Stupkata na Bogoroditsa, 9—Sharapanite, 10—Palaeokastro, 11—Gaydarov Dolap, 12—Blaga Cherkva, 13—Ponichkata, 14—Evdzhika, 15—Kalinkin Kamuk, 16—Kamennata Kushta.

Sampling was carried out between 28 and 30 June 2023 in the Haskovo district (Southeast Bulgaria) on seven granite megaliths in the vicinity of the town of Topolovgrad (Table 1, Figure 1). The names of these historical monuments were transliterated according to the transliteration law of the Bulgarian government [53]. The classification of the megalith types and the terminology used are the same as in our earlier study [45].

**Figure 2:**
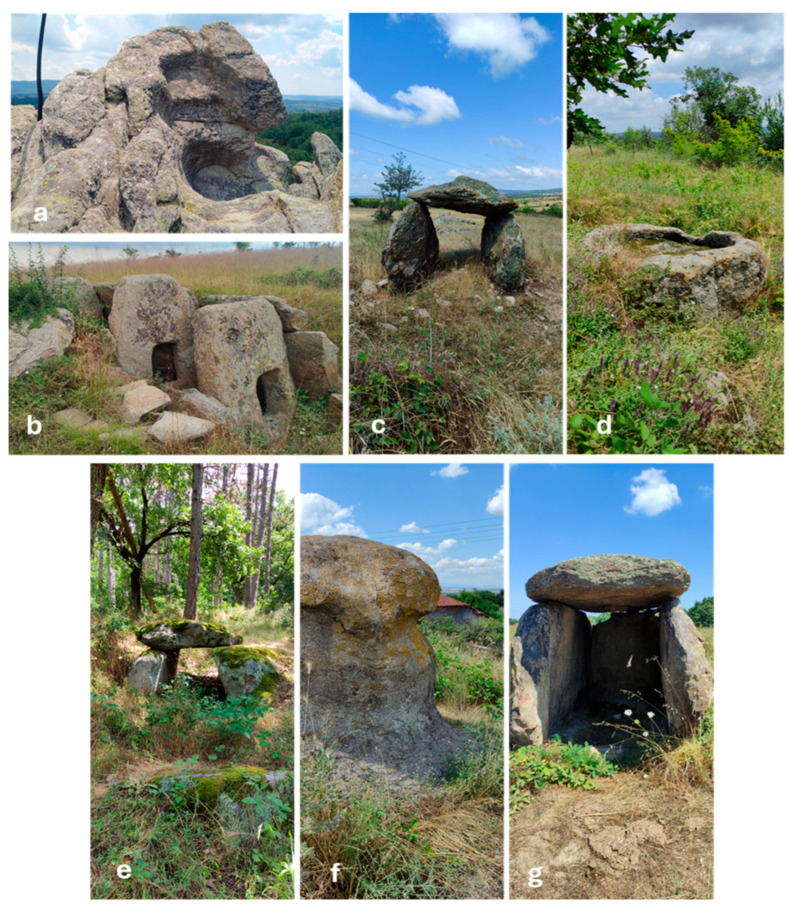
Investigated megaliths in the region of Topolovgrad, Haskovo district: (**a**)—Part of Palaeokastro Thracian cult and fortress complex, (**b**)—Double-chambered dolmen Gaydarov Dolap, (**c**)—Dolmen Blaga Cherkva, (**d**)—Ponichkata, (**e**)—Protodolmen Evdzhika 2, (**f**)—Kalinkin Kamuk, (**g**)—Dolmen Kamennata Kushta.

The investigated sites (Figure 2a–g) include a megalithic sun cult and fortress complex (i.e., Paleocastro) and six separate megaliths (four dolmens and two single stones)—Figure 2a. Five of them are located in open areas, with the exception of the protodolmen Evdzhika 2, which is situated in a shady place in a mixed deciduous forest, and Ponichkata, which is surrounded by deciduous trees on its western side (Figure 2d). Six of the seven investigated sites are located in isolated and unpopulated areas, and only Kalinkin Kamuk is located within the village of Hlyabovo (Figure 2f). The surfaces of all megaliths are densely covered with crustose lichens and only the surfaces of Evdzhika 2 are mainly covered with mosses (Figure 2e).

The temperature of the megalithic surfaces (Table 1) was measured directly with the Bosch GTC 400C thermal camera (Robert Bosch Power Tools GmbH, Stuttgart, Germany) with a measuring range of −10 to +400 °C. Since we observed the megaliths simultaneously with a DJI Mavic 2 Enterprise Dual Pro (DJI Technology Co., Ltd., Shenzhen, China) drone equipped with a photo and thermal camera, the surface temperature data obtained from the drone were compared with those from the direct measurement.

**Figure 3:**
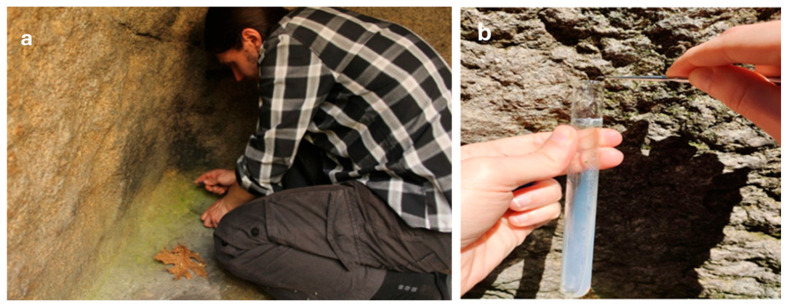
Example of sampling from a visible algal layer (**a**) and inoculation of the material in a test tube with an inclined agar (**b**) from the megalithic surfaces using the direct collection method [54].

Following the Direct Collection Method [54], 26 mixed qualitative algal samples (Table 1) of each layer with visible coloration were scraped from the megalithic surfaces on slant agar covered with Bold Basal Medium—BBM in glass test tubes (Figure 3). In this way, the stone-associated aero-terrestrial algae that develop on rock surfaces and are commonly known as epilithic algae (epiliths) [1,8,55,56], were collected. About 0.5–1 cm^2^ of each visible layer was scraped off, depending on its size. By finely scoring the surface layers with a medical scalpel or dental needle, no rock surface was affected, ensuring that none of the ancient monuments were damaged. To avoid any contamination, the scalpel or needle was cleaned with hydrogen peroxide (H_2_O_2_) or ethanol (70%) before and after each inoculation.

After collecting, the samples were transported to the laboratory, where the material was processed for further cultivation on BBM agar and clone cultures were obtained according to standard methods [54,57]. Subsequently, each sample was analyzed at regular intervals according to the visible growth of the culture. From each visible colony material was taken by a dental needle for the preparation of non-permanent microscopic slides and, if necessary, the preparation of slides was doubled or tripled. In this way, 356 microscopic slides were analysed. The algae were identified under an Olympus BX53 light microscope (Olympus Corporation, Tokyo, Japan). The microphotos were taken with the Olympus DP72 photomicrograph camera (Olympus Corporation, Tokyo, Japan).

The taxonomic sources used for the identification of the algae include the widely used European taxonomic floras and manuals (e.g., [1,58,59,60,61,62,63,64,65,66,67,68,69,70,71,72]) with the currently published relevant works. Taking into account the International Code of Nomenclature of Algae, Fungi and Plants [73,74], the phylum name Cyanoprokaryota [58,59,60] is used for the prokaryotic blue-green algae, also known as cyanobacteria. All other updates to the synonymy, as well as the distribution data, follow AlgaeBase [75]. The new algae for Bulgaria were checked according to [76]. The conservation status of the recorded species followed the Red List of Bulgarian Microalgae [77]. The floristic similarity of the megaliths was estimated using Sørensen Similarity Index (SSI) [78], widely used in botany, to allow comparison with the similarity data obtained in our earlier study on nine megaliths in the same district of Haskovo [45]. The potential toxin producers were determined from the available data on toxic genera and species (for details see [79,80,81,82,83]). The correlation coefficient r from Microsoft Excel version 2406 for Windows 11 was estimated and if the statistical threshold was at least *p* < 0.05, the correlation was accepted as significant [84]. In agreement with our previous study [45], correlations were estimated between the total algal biodiversity (represented by the number of species) at each site and the corresponding temperatures, between the total diversity and the lowest and highest temperatures of each megalith, and between all these temperatures and the number of species in the different algal divisions. In addition, a statistical analysis was performed based on the Non-metric MultiDimensional Scaling (NMDS) technique [85,86,87] using the statistical language R (v4.5.1; R Core Team, 2025).

## 3. Results

**Figure 4:**
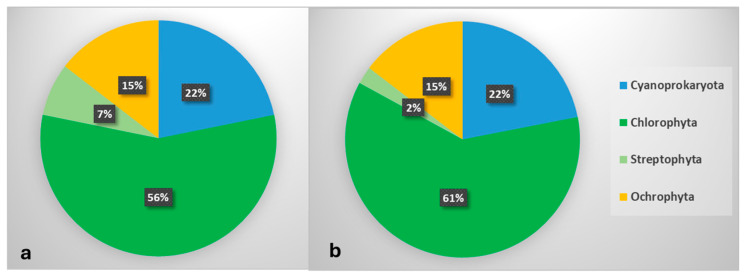
The overall biodiversity of algae in the seven megaliths in the Haskovo district, represented as the number of species (**a**) and the number of genera (**b**) in the main taxonomic groups. The numbers in the black boxes show the relative contribution of each division to the overall taxonomic structure.

A total of 55 species from 41 genera of four divisions were identified: Cyanoprokaryota (12 species from 9 genera), Chlorophyta (31 species from 25 genera), Streptophyta (four species from one genus), Ochrophyta (8 species from 6 genera)—Figure 4, Table 2.

Most species were only rarely distributed (Table 2): 34 of them, i.e., 62%, were found on only one megalith, 14 (25%) on two megaliths, three (5%) on three megaliths and two (4%) on four megaliths. Only two species were more widespread and were found on seven megaliths (*Stichococcus bacillaris*—and six megaliths (*Mychonastes homosphaera*).

In addition to the common and widespread aeroterrestrial species, we also found some rare algae that occur mainly in the northern, polar or alpine regions of the world, such as *Phormidium calcareum*, *Diplosphaera chodatii*, *Lobosphaera incisa*, *Macrochloris radiosa*, *Pseudodictyochloris multinucleata*, *Pseudomuriella engadinensis* and *Vischeria helvetica* [1,8,75].

Two of the recorded species are of conservation importance, namely *Coelastrella aeroterrestrica* and *Vischeria stellata*, which are considered critically endangered and endangered in the Red List of Bulgarian microalgae [77].

Six species from the cyanoprokaryotic genera *Aphanocapsa*, *Aphanothece* and *Leptolyngbya* (Table 2) were listed among the toxin producers [79,80,81,82,83].

**Figure 5:**
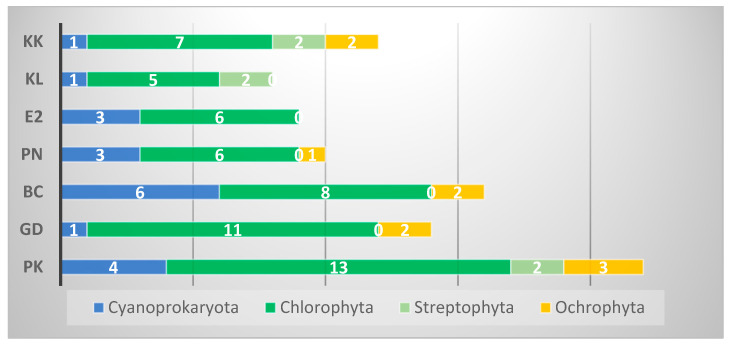
General algal diversity in each of the seven investigated megaliths, represented by the number of species in each of the main taxonomic groups (white numbers). The abbreviations on the ordinate denote the megaliths: PK—Paleokastro, GD—Gaydarov Dolap, BC—Blaga Cherkva, PN—Ponichkata, E2—Evdzhika 2, KL—Kalinkin Kamuk, KK—Kamennata Kushta.

The number of species detected in the individual megaliths varied between 8 and 22 (Table 2, Figure 5). The richest species composition (22 species) was found in the large ancient sun cult complex Palaeokastro, while the lowest number of algae (8 species) inhabited the single megalith Kalinkin Kamuk.

**Figure 6:**
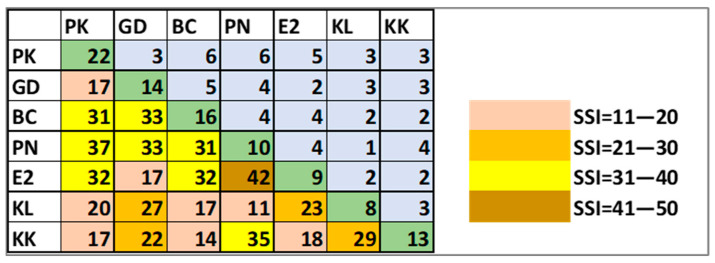
The floristic similarity between the seven megaliths analyzed is represented by the Sørensen’s Similarity Index (SSI) [78]. The number of species in each megalith is shown with bold numbers on the diagonal (green). Above the diagonal, the number of common species for each two megaliths is shown (light blue), and below the diagonal the values of the SSI are shown, categorized into four classes up to 10%. The abbreviations denote the megaliths: PK—Paleokastro, GD—Gaydarov Dolap, BC—Blaga Cherkva, PN—Ponichkata, E2—Evdzhika 2, KL—Kalinkin Kamuk, KK—Kamennata Kushta.

The floristic similarity estimated by the SSI between the analyzed megaliths was low (11–42%)—Figure 6. The index has the highest value of 42% only between Ponichkata and Evdzhika 2 and the lowest value of 11% between Ponichkata and Kalinkin Kamuk. The second relatively high similarity of 37% was between Ponichkata and Paleokastro. The other SSI values ranged between 11 and 35%.

Considering that all megaliths analyzed have a common granitic character, the highest value of the SSI of 42% between Ponichkata and Evdzhika 2 could be explained by their location in less exposed, shadier places under or near trees. In this case, the reason for the lack of greater similarity could be sought in the significant temperature differences (17–18.3 °C on the surfaces of Evdzhika 2 and 25.3–31.3 °C on the surfaces of Ponichkata—Table 1) and in the different organisms overgrowing the rocks—crustose lichens on Ponichkata (Figure 2d) and mosses on Evdzhika 2 (Figure 2e). The greater distance from heavily populated areas could explain the relatively high SSI (37%) between the small, single megalith Ponichkata and the large cult complex Paleokastro (Figure 6). This is supported by the very low value of SSI (11%) between two single stones—Ponichkata and Kalinkin Kamuk (Figure 6). In this last case, exposure and location also seem to play a role, because unlike Ponichkata, Kalinkin Kamuk (Figure 2f) is located in a sunny, highly exposed place in a village, close to the tarmac road.

**Figure 7:**
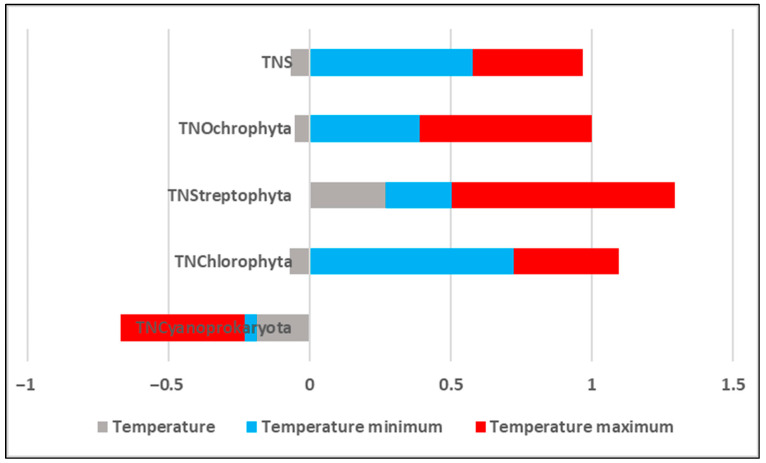
Values of correlation coefficients estimated as correlations between the temperatures of the seven megaliths analyzed (minimum and maximum for each megalith and by sample) and algal biodiversity (total and by taxonomic division). TNS—total number of species, TN combined with the name of the phylum—total number of species of the algal division in question.

The correlations between the total algal diversity, represented as the total number of species, at each sampling point and the corresponding temperatures at these points, as well as the total diversity in each division, were negative and very low (r = 0.1–0.2). The exception was the relatively higher and positive correlation between temperature and the number of species in the Streptophyta (r = 0.3)—Figure 7.

The diversity of these algae, represented in this study only by the genus *Klebsormidium* (Table 2), was also strongly correlated with the highest measured temperature for each megalith (r = −0.8) but only weakly correlated with the minimum temperatures (r = 0.2).

In contrast, the diversity of the other green division, Chlorophyta, was strongly correlated with the minimum temperature (r = 0.7) and less correlated with the maximum temperature (r = 0.4). For Ochrophyta, r = 0.4 and r = 0.6 were estimated for minimum and maximum temperature, respectively. Similarly, albeit much weaker and negative, the estimated correlations for the phylum Cyanoprokaryota were r = −0.04 with the lowest temperatures and r = −0.4 with the maximum temperatures (Figure 7).

**Figure 8:**
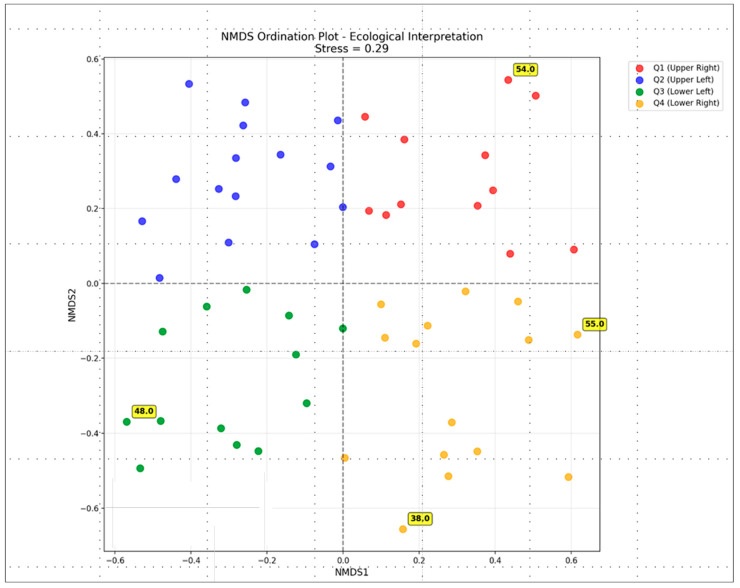
NMDS ecological interpretation plot where each point represents a species and the NMDS1 and NMDS2 axes represent the primary gradients of variation. Species are colored by quadrant to show four ecological groupings (Q1, Q2, Q3, Q4). The outlier species that represent unique ecological positions are indicated with their numbers from the original dataset -for details see the text of the paper.

The NMDS analysis, based on all species and environmental parameters using Bray–Curtis similarity as a distance metric, was completed in the 2D ordination space with a stress value of 0.29 (fair interpretation); the main resulting diagram is shown in Figure 8.

**Figure 9:**
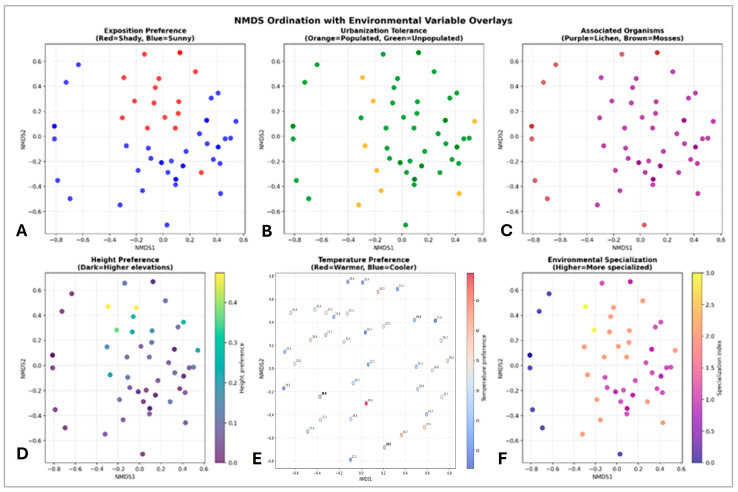
Comprehensive NMSD ordination analysis with the following environmental overlays: (**A**) exposure preferences (shady/sunny), (**B**) urbanization tolerance (populated/unpopulated areas), (**C**) associated organisms (lichens/mosses), (**D**) height preference, (**E**) temperature preference and (**F**) environmental specialization. There is a corresponding legend for each diagram.

The ecological interpretation of NMDS results (Figure 9) demonstrates how all 55 species are positioned in relation to each other based on their ecological similarities. The arrangement of species in the four color-coded quadrants suggests that species group together based on similar environmental preferences or habitat requirements, indicating four distinct ecological niches and four main relevant ecological groups. The distribution of species across all four quadrants indicates considerable ecological variation among the species. The pattern of species spread by quadrant (Figure 9) shows that the first group (Q1, top right) comprises 11 species, the second group (Q2, top left) 15 species, the third group (Q3, bottom left) 14 species and the fourth group (Q4, bottom right) 15 species. The four species marked as extreme (numbers 38, 48, 54, 55 in Figure 8) represent unique ecological positions, which may be specialized species with particular habitat requirements, species with unusual environmental tolerances or potentially rare species or indicator species. These species are *Stichococcus mirabilis* (38), *Navicula* sp. (48), *Nephrodiella minor* (54) and *Pleurochloris commutata* (55).

The further NMDS for all 55 species analyzed across the environmental gradients shows a moderate stress value of ~60.5 (Figure 9). It was found that 44 species prefer sunny conditions versus 15 preferring shade, 42 species tolerate unpopulated areas versus 8 in populated areas, 43 species are associated with lichens versus 10 with mosses, and that there is a continuous gradient in the spread of species regarding the elevation of the studied sites.

**Figure 10:**
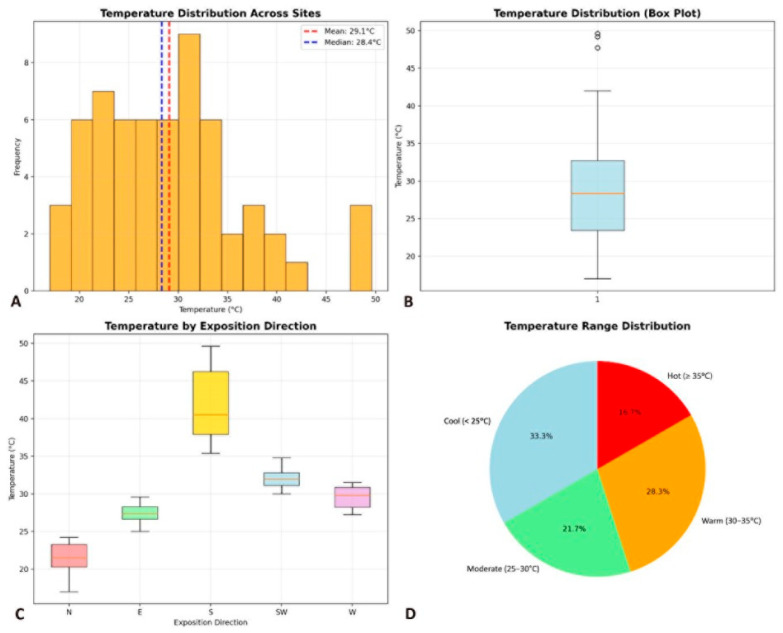
Diagrams of NMDS analysis in terms of temperature and exposition direction: (**A**)—Distribution of species frequency in sites of the whole temperature range; (**B**)—Box plot with distribution of sites by temperature: (**C**)—distribution of sites in terms of temperature and exposition direction (N—North, E—East, S—South, SW—South-West, W—West); (**D**)—Distribution of species in sites of different temperature range.

In terms of exposition direction and temperature (Figure 10A–D), the epilithic species during this study were found at sites with a wide range of temperatures from 17.0 °C to 49.6 °C with a mean of 29.1 °C (Figure 10A). Most sites were with temperatures between 24 and 33 °C (Figure 10B). The distribution of species by their frequency was fairly well spread over different thermal categories: The largest group of species (28%) was found at warm sites (30–35 °C), while at cool sites (<25 °C) one third of the species (33%) were found, at moderate temperature sites (25–30 °C) about one fifth (22%) of the species occurred and the smallest number of species (17%) was found at hot sites (≥35 °C)—Figure 10A,D. Most sites with high temperatures were the sites of southern exposure and the coolest were the sites with northern exposure (Figure 10C).

**Figure 11:**
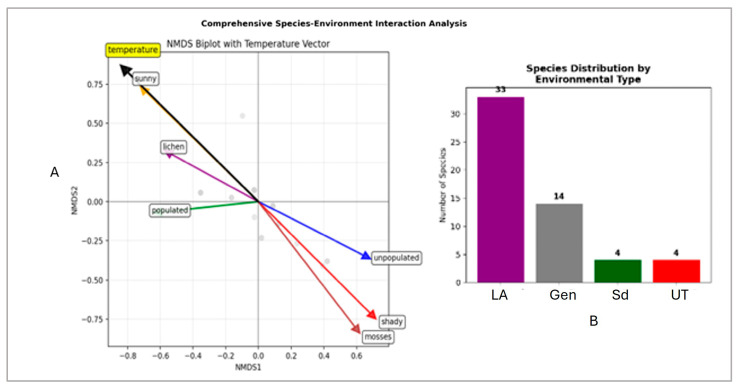
Comprehensive NMDS analysis: (**A**)—map of species-environment interaction with environmental vectors; (**B**)—distribution of species by environmental type: LA—lichen associated, Gen—generalists, Sd—shady, UT—urban-tolerant species.

A further comprehensive NMDS species-environment interaction analysis revealed several important ecological patterns. In terms of environmental specialization, sun specialists (32 species) dominate over shade specialists (4 species), urban avoiders (39 species) prevail over urban-tolerants (4 species), and lichen-associated species are most abundant (37 species) over moss-associated species (3 species). The same analysis made it possible to outline the significant environmental gradients (*p* < 0.001)—Figure 11A. The dominant axis, temperature and sunny exposure versus shady exposure, shows that these factors are the most important environmental vectors determining the distribution of epilithic species. Temperature is consistent with sunny and in contrast to shady/mossy sites, meaning that warmer, sunnier assemblages differ from cooler, shady, mossy ones. Species located near these arrows are associated with these conditions and do not usually co-occur. The lichens, some of which correspond to sunny habitats and higher temperatures, show a modest association in the upper left quadrant. The unpopulated vector aligns away from the populated one. This suggests a secondary gradient related to human presence/absence, but its influence appears to be weaker than temperature and light vectors. In addition, based on the clustering of species four main groups can be distinguished (Figure 11B). The largest of these is the group of lichen associates, followed by the generalists with moderate environmental requirements and two small but distinct groups of shade specialists and urban-tolerants.

## 4. Discussion

This study revealed a rich algal flora with a total of 55 algal species from four divisions identified on the surfaces of the seven megaliths near the town of Topolovgrad in the Haskovo district of southeastern Bulgaria. A comparison of these results with the algal biodiversity of nine other megaliths throughout the Haskovo district, which we had analyzed in a previous study [45], showed that 20 (36%) of the species were detected for the first time from surfaces of Bulgarian megaliths. Referring to the currently compiled checklist of Bulgarian lithophytic algae [88] and to the recently published work on algae from a granite monument in Sofia [89], 15 species identified during this study were newly recorded for this ecological group in the country. Moreover, 14 of them were reported for the first time for the Bulgarian algal flora.

Although different taxonomic approaches have been applied and most data on megaliths concern their moss and lichen flora [48,94,95], it can be stated that algal diversity is richer on the studied megaliths than on other megaliths and historical granite monuments [29,90,91]. This result is the more interesting when one considers that the comparison of algal biofilms on historical monuments from different lithotypes showed that granite is less colonized by blue-green algae and green algae than marble and limestone [29]. The general number of species found and the prevalence of Chlorophyta (55 and 31 species, respectively) recorded on the megaliths during the hottest summer months largely corresponds to the biodiversity of epilithic algae on the granite walls of various Ukrainian river gorges [93] and, especially to their diversity in a river gorge in the forest zone of Ukraine (69 species, 72% of which are chlorophytes), which is wetter and shadier than the area we studied [92]. At the same time, the diversity of epilithic cyanoprokaryotes found on the studied megaliths is much greater (12 species from nine genera) compared to other granite historical monuments or natural rocks, on which up to three species from this division were found [90,91,92].

The taxonomic structure resulting from this study is consistent with the general prevalence of green algae and cyanoprokaryotes as common epilithic colonisers often expressed by various authors (e.g., [26,29,45,76,96,97,98]). In terms of detailed species composition, most species were common and widespread aeroterrestrial inhabitants, but seven chlorophyte algae were rarely reported, mainly from different, colder regions of the world. Their occurrence in the highly sun-exposed habitats of southeastern Europe therefore expands our knowledge of their ecological requirements and indicates their greater tolerance.

Considering the conservation importance of the recorded species, two of them are on the Red List of Bulgarian microalgae [77], but according to the proposed methodology for assessing the endangered status of microalgae [99], most of the newly included algae for the country, and, in particular, the seven species discussed above, could be proposed for inclusion in this list.

Epilithic algae grow on surfaces exposed to the atmosphere; they are often transported by air currents and can be aerosolized by raindrops during precipitation [80]. Such aerosols can easily enter the human organism and cause an allergenic reaction when inhaled or deposited on the skin [79,80,81]. In addition to these ways of infecting humans, there is another form of direct contact via the skin when it rubs against a surface colonized by algae [80]. It has already been suggested that the built environment may suffer from more and larger amounts of harmful algal species in the future due to global warming [80]. Therefore, we emphasise here that among the 55 algae identified, six cyanoprokaryotes from the genera *Aphanocapsa*, *Aphanothece* and *Leptolyngbya* are known for their toxin-producing potential [79,80,81,82,83] and could therefore pose a potential threat to the health of visitors to megaliths.

Most algae were rarely distributed, with 51 species (93%) recorded from one to three megaliths, in contrast to the four species that were distributed at more sites (Table 2). Only one species, the green *Stichococcus bacillaris*, was found as an epilith on all seven megaliths. This filamentous green alga is generally widespread throughout the world in various habitats, including natural granitic rocks [92,93] and historical monuments and buildings, and has a high drought tolerance [1,8,26,29,91,100,101]. The same physiological characteristic explains the relatively wide distribution of the green coccal *Mychonastes homosphaera* on six megaliths. This species has been shown to have the ability to live in soils and even in deserts [102,103], and to exhibit an epilithic lifestyle on natural rocks [92] and historic buildings [26]. Both species were detected in our earlier study of megalithic surfaces, and *Stichococcus bacillaris* was more widespread compared to *Mychonastes homosphaera* [45]. The additional advantage of *Streptococcus bacillaris* in the colonization of megalithic surfaces could lie in its allelopathic abilities against mosses [80,104], which can also colonise megalithic surfaces, as in the case of Evdzhika 2.

The rare occurrence of algae on the megaliths studied is consistent with the low floristic similarity (11–42%) between the sites. The fact that all the megaliths studied have a common granitic character, despite their different size, location and exposure, points to the importance of microclimatic effects and autecology in understanding the algal colonization of megalithic surfaces, most of which are exposed to strong solar radiation and drought. Similar conclusions from our earlier study [45] are consistent with the statement that colonization by cyanoprokaryotes and green algae depends primarily on the physical characteristics of the stone surfaces, microclimate and environment, and only secondarily on the lithotype [29]. Together with the lowest number of species, the lowest similarity of Kalinkin Kamuk, which is located in a populated area near a road, with the other six megaliths allows us to hypothesise the influence of air quality on the diversity of the megalithic flora studied. The role of air pollution and even air eutrophication in the distribution and development of aeroterrestrial algae has already been shown to be important despite the few studies conducted on this topic [26]. The comparison of the results of this study with the similarity between nine other megaliths from the same district of Haskovo suggests that the distance between the analyzed sites plays a role, as the SSI values were generally higher for the seven closer megaliths (11–42%, in eight cases the SSI was between 31 and 37%) than for the nine megaliths scattered in different parts of the district, where the SSI was 0–33%, in most cases being 12–18% [45].

The limited distribution of most of the identified algae is consistent with the results of our previous investigations of algae on various historical and natural rock monuments in Bulgaria [45,76,96,97,98]. Especially in view of the previously obtained results on the megaliths [45], this was the second reason to continue the search for temperature dependence, through correlations between temperature and its extremes with algal biodiversity expressed by total number of species and number of species by division and to try to outline the species most likely to survive global warming. This work was undertaken due to the significant threat that climate change poses to historical monuments [48]. The effects on substrates and epilithic algae are due not only to rising temperatures and their extremes, but also to the associated decrease in precipitation and increase in the intensity and duration of drought (e.g., [105,106]). It should be noted that the results obtained are still preliminary due to the generally small number of sites studied and do not fully agree with the correlations we estimated for nine other megaliths, where maximum temperatures were much higher, i.e., 42–49.6 °C, and more surfaces with temperatures above 30 °C occurred [45]. We must also bear in mind that studies on algae as indicators of climate change are still scarce and are based only on recent works at a few, mostly aquatic, sites, which makes any quantification quite difficult without certain prior knowledge [107]. However, according to the results of this study, in which the Streptophyta showed the highest positive correlation with maximum temperatures, it can be assumed that most of the lithophytic survivors may be found among these algae as warming increases. Combining the results of the correlation coefficients and the distribution of species also shows that the species with the best chances belong to the genera *Klebsormidium* (Streptophyta), which are among the algae that dominate natural granitic rocks [92,93] and are known for their adaptability to strong sunlight, temperature extremes and drought (e.g., [12,26,108,109,110]), as well as the above-mentioned *Stichococcus* (Chlorophyta). Although all four *Klebsormidium* species identified in this study were sparsely distributed, they were found on the most exposed surfaces with the highest temperatures of Paleokastro, Kalinkin Kamuk and Kamennata Kushta. These results are consistent with the data we obtained in a previous study [45] on both genera, in which we already summarized the literature data and emphasized their ability to withstand unfavorable conditions related to extreme temperatures, drought and strong solar radiation.

In support of the perspective of further studies on *Stichococcus* species, we must note that *Stichococcus mirabilis* was one of the four species labelled as extreme in the NMDS ordination plot. Together with three other species (i.e., *Navicula* sp., *Nephrodiella minor* and *Pleurochloris commutata*), it occupied the outermost unique positions in this plot and should therefore be further investigated as a potential indicator species with regard to their particular habitat requirements or unusual environmental tolerances.

The NMDS ordination clearly shows that the species found on the megaliths exhibit considerable ecological differences in terms of their environmental preferences. It successfully separates the epilithic species with different ecological strategies into four distinct ecological groups of lichen associates, generalists, shade specialists and urban-tolerants. Furthermore, NMDS analysis of environmental vectors based on environmental overlays revealed that temperature and light are the strongest factors for species dispersal on megalithic surfaces.

## 5. Conclusions

This study shows that megaliths (ancient stone structures) can harbour a rich algal flora with a variety of species of green algae, cyanoprokaryotes, and others that thrive in harsh environmental conditions. In this respect, the recent discovery of some rare chlorophyte species on highly exposed surfaces in southeastern Europe, mainly reported from northern countries, polar and alpine regions, is of particular importance and indicates their greater ecological tolerance. Together with the proven differences in the species composition on megaliths from the same region and the different ecological strategies of the epiliths, this shows the need for future more detailed investigations, reinforced by a polyphasic approach to this as yet untaped biodiversity. The discovery of potential toxin producers among the species on the surfaces of the megaliths is worrying for the health of tourists and requires further studies on harmful strains and their toxins. Understanding the diversity of algae on megaliths contributes to the knowledge of the biology and ecology of lithophytic algae but is also important for the conservation and for understanding the impact of climate change and other factors on these fragile, highly susceptible monuments. The application of the NMDS analytical tool showed that the most important drivers for the distribution of the epilithic algae on the investigated megaliths are their temperature and light preferences. Although there are no data on their past algal diversity, the combined results on species distribution and correlations between algal composition and temperatures allowed us to hypothesise the importance of various green algae, and, in particular, the filamentous *Klebsormidium* (Streptophyta) and *Stichococcus* (Chlorophyta), as future epilithic survivors under conditions of increasing warming and drought.

## Figures and Tables

**Table 1:** Names and locations of the seven selected megaliths from the Topolovgrad region in the Haskovo district, with their abbreviations (Abb), geographic coordinates (GC), the temperature range (TR [°C]), the exposure (Exp), the range of the height over the earth surfaces (HR [cm]), dominant associated organisms (DAO) and exposition direction (N—north, E—east, S—south, SW—southwest, W—west) of the sites from which the algae were collected and the number of samples taken (NCS).

Megalith	Abb	GC	TR *	Exp	HR	DAO	ED	NCS
Paleokastro	PK	42°04′46.8″ N, 26°18′05.6″ E	31.4–40.3	Sunny	25–260	Lichens	N, E, S, SW	7
Gaydarov Dolap	GD	42°02′48.2″ N 26°14′14.3″ E	27.4–32.3	Sunny	20–60	Lichens	E, SW, W	4
Blaga Cherkva	BC	41°57′43.9″ N 26°08′13.9″ E	20.4–23.6	Sunny	25–90	Lichens	E, S, W	4
Ponichkata	PN	42°03′09.8″ N 26°16′03.8″ E	25.3–31.3	Shady	35–65	Lichens	E, W	2
Evdzhika 2	E2	42°02′38.5″ N 26°15′30.6″ E	17–18.3	Shady	8–10	Mosses	N, S	2
Kalinkin Kamuk	KL	42°03′14.6″ N 26°15′12.7″ E	23.5–34.8	Sunny	160–180	Lichens	E, SW, W	3
Kamenna Kushta	KK	42°02′20.6″ N 26°10′16.0″ E	19.6–40.7	Sunny	20–75	Lichens	E, S, W	4

* The temperature data are original empirical measurements obtained during sampling.

**Table 2:** Species composition of the seven investigated megaliths from Topolovgrad region of Haskovo district, southeastern Bulgaria: PK—Paleokastro, GD—Gaydarov Dolap, BC—Blaga Cherkva, PN—Ponichkata, E2—Evdzhika 2, KL—Kalinkin Kamuk, KK—Kamennata Kushta. Within the divisions, the species are arranged in alphabetical order. The species listed as Critically Endangered (CR) or Endangered (EN) in the Red List of Bulgarian Microalgae (RLBM) [77] are noted. Potentially toxic (PT) species are shown following [79,80,81,82,83]. Previous records of each species from the megaliths (M) or from other substrates (O) in the lithophytic flora of Bulgaria (PRLFB) [45,88,89] and from other granite monuments (G) or natural rocks (R) in the world (GW) [29,90,91,92,93] are indicated. New species for the country are marked with an asterisk (*).

Species/Megaliths	PK	GD	BC	PN	E2	KL	KK	RLBM	PT	PRLFB	GW
CYANOPROKARYOTA											
*Aphanocapsa* sp. 1			x						+	M	
*Aphanocapsa* sp. 2	x			x					+	M	
*Aphanothece* sp. 1	x		x		x	x			+	M	
*Aphanothece* sp. 2		x	x						+	M	
* *Aphanothece* sp. 3				x			x		+		
*Gloeobacter violaceus* Rippka, J. B. Waterbury et Cohen-Bazire	x			x						M	
*Leibleinia epiphytica* (Hieronymus) Compère					x					M ^1^	
*Leptolyngbya compacta* Komárek					x				+ ^2^	M ^3^	
* *Phormidium calcareum* Kützing ex Gomont			x								
* *Pseudophormidium spelaeoides* (Cado) Anagnostidis			x								
* *Synechocystis primigenia* N. L. Gardner	x										
*Trichocoleus delicatulus* (West & G. S. West) Anagnostidis			x							O	
CHLOROPHYTA											
*Apatococcus lobatus* (Chodat) Petersen							x			M, O	G,R
*Chlorella miniata* (Kützing) Oltmanns		x		x			x			O	
*Chlorella vulgaris* Beijerinck		x								M, O	G
*Chloroidium ellipsoideum* (Gerneck) Darienko, Gustavs, Mudimu, Menendez, Schumann, Karsten, Friedl et Proschold	x				x					M, O	R
*Choricystis parasitica* (Brandt) Pröschold et Darienko		x								M, O	
*Coelastrella aeroterrestrica* Tschaikner, Gärtner et Kofler	x		x					CR			
*Coelastrella striolata* Chodat						x					
*Coenobotrys gloeobotrydiformis* (Reisigl) Kostikov, Darienko, Lukesová et Hoffmann		x								M, O	
*Desmococcus olivaceus* (Persoon ex Acharius) Laundon	x			x						M, O	G,R
*Deuterostichococcus tetrallantoideus* (Kol) Pröschold et Darienko	x									M	
* *Diplosphaera chodatii* Bialosuknia			x								
*Edaphochlorella mirabilis* (Andreeva) Darienko et Pröschold	x		x	x	x					M, O	
*Elliptochloris reniformis* Darienko et Pröschold	x		x							O	
* *Lobosphaera incisa* (Reisigl) Karsten, Friedl, Schumann, Hoyer et Lembcke			x								
*Lobosphaeropsis lobophora* (Andreeva) Ettl et Gärtner		x								M, O	
* *Macrochloris radiosa* Ettl & Gärtner							x				
*Muriella decolor* Vischer	x									M	
* *Muriella magna* F. E. Fritsch et R. P. John							x				
*Muriella terrestris* J. B. Petersen		x				x				M	
*Mychonastes homosphaera* (Skuja) Kalina et Puncochárová	x	x	x	x	x		x			M, O	G,R
*Neocystis brevis* (Vischer) Kostikov et Hoffmann	x	x				x				M	
*Parachlorella kessleri* (Fott et Nováková) Krienitz, Hegewald, Hepperle, Huss, Rohr et Wolf	x									M	
*Pseudodictyochloris multinucleata* (Broady) Ettl & Gärtner		x								M, O	
* *Pseudomuriella engadinensis* (Kol et F. Chodat) Fuciková, Rada et L. A. Lewis				x	x						
*Pseudostichococcus monallantoides* var. *exiguus* (Gerneck) Pröschold et Darienko	x									M,O	
*Sphaerococcomyxa olivacea* (Petersen) Kostikov, Darienko, Lukesová et Hoffmann.							x			M	
*Stichococcus bacillaris* Nägeli	x	x	x	x	x	x	x			M, O	G,R
*Stichococcus minutus* Grintzesco et Ș. Péterfi	x									M, O	
*Stichococcus mirabilis* Lagerheim		x	x							M	
*Tetracystis pulchra* R. M. Brown et Bold					x					M	
* *Ulothrix implexa* (Kützing) Kützing						x					
STREPTOPHYTA											
*Klebsormidium crenulatum* (Kützing) Lokhorst	x									M	R
*Klebsormidium dissectum* (Gay) Ettl et Gärtner						x	x			M, O	
*Klebsormidium flaccidum* (Kützing) Silva, Mattox et Blackwell						x	x			M, O	G,R
* *Klebsormidium montanum* (Hansgirg) Shin Watanabe ex Molinari et Guiry	x										
OCHROPHYTA											
* *Ellipsoidion parvum* H. Reisigl			x								
*Ellipsoidion perminimum* Pascher		x	x	x						M	
*Gloeobotrys ellipsoideus* Pascher	x									O	
*Navicula* sp. s.l.							x			M, O	
* *Nephrodiella minor* Pascher	x										
*Pleurochloris commutata* Pascher		x								M	
*Vischeria helvetica* (Vischer et Pascher) D. J. Hibberd	x						x				
*Vischeria stellata* (Chodat) Pascher							x	EN		M, O	

^1^ As “*Leptolyngbya* sp. 4 (? *Leiblenia* sp.); ^2^ Considering data on the potential toxicity of the genus *Leptolyngbya*; ^3^ As “*Leptolyngbya* sp. 1 (ad *Leptolyngbya compacta* Komárek)”.

## Data Availability

The original contributions presented in this study are included in the article material. Further inquiries can be directed to the corresponding author.
